# Effectiveness of Regular Aerobic Exercise in Improving Vascular Stiffness in Elderly Korean Women

**DOI:** 10.3390/jcm12196119

**Published:** 2023-09-22

**Authors:** Suhan Koh, Taekyu Kim, Duwang Kang, Doyeon Kim

**Affiliations:** Department of Physical Education, Pusan National University, Busan 46241, Republic of Korea

**Keywords:** aerobic exercise, elderly Korean women, arterial stiffness, cell adhesion molecule, oxidized LDL

## Abstract

The purpose of this study was to determine the effects of aerobic exercise on carotid to femoral pulse wave velocity (cf-PWV), cell adhesion molecules (intracellular adhesion molecules (ICAM-1), vascular cell adhesion molecules (VCAM-1), endothelial selectin (E-selectin), and oxidized LDL in elderly women aged 70–85 years, and to identify the effect of and correlation with vascular stiffness. Forty participants were recruited and divided into three groups; vascular stiffness (VSG, *n* = 14), obesity (OG, *n* = 14), and normal (NG, *n* = 12). All groups were given a 16-week intervention of aerobic exercise, and the data collected before and after exercise were analyzed using SPSS Ver. 23.0. Two-way repeated-measures ANOVA was used to evaluate between-group and time-dependent interaction effects. One-way ANOVA was used to evaluate between-group variations. In addition, the significance was tested using a post hoc test (*Scheffe*). The within-group variations by time before and after exercise were examined using a paired *t*-test, and correlation analysis was performed using Pearson correlation coefficients. Simple regression analysis was performed for variables showing significant differences. The results indicate interaction effects for cf-PWV (*p* < 0.001), VCAM-1 (*p* < 0.01), E-selectin (*p* < 0.05), and oxidized LDL (*p* < 0.001). The rate of change of cf-PWV was positively correlated with that of VCAM-1 (*r* = 0.352, *p* < 0.05) and that of oxidized LDL (*r* = 0.325, *p* < 0.05) with statistical significance. To determine the effect of the rate of change of cf-PWV on the rate of change of VCAM-1, the variables were tested, and the coefficient of determination in the regression analysis was 0.124, indicating that 12.4% of the tested variables fit the standard regression line. The variables for the effect of the rate of change of cf-PWV on the rate of change of oxidized LDL were also tested, and the coefficient of determination in the regression analysis was 0.106, indicating that 10.6% of the tested variables fit the standard regression line. Thus, the 16-week regular and consistent aerobic exercise program had significant effects on the cf-PWV, ICAM-1, VCAM-1, E-selectin, and oxidized LDL in elderly Korean women with vascular stiffness, suggesting improvements in vascular stiffness, based on which the intervention is predicted to contribute to the prevention of vascular dysfunction by lowering the risk of cardiovascular disease due to atherosclerosis, as well as having a positive effect in the prevention of impairment of vascular endothelial cells.

## 1. Introduction

Cell adhesion molecules (CAMs) are expressed in vascular endothelial cells as glycoproteins that can receive the signals of IL-1/TNF-α and IL-8 secreted by macrophages to drive leukocytes in peripheral blood to adhere to vascular endothelial cells, allowing them to flow into damaged tissues.

However, in designing the present study, it was presumed that aging in elderly women could lead to vascular dysfunction and impairment of vascular endothelial cells due to the overexpression of CAMs. The previously positive roles of CAMs in normal defense mechanisms of the body could exert a negative effect on vascular and metabolic functions. Nevertheless, a recent study reported that certain types of CAMs, intracellular adhesion molecule-1 (ICAM-1) and vascular cell adhesion molecule-1 (VCAM-1), were overexpressed even in the early stage of atherosclerosis [1], and a high level of endothelial selectin (E-selectin) increased the risk of metabolic syndrome to a greater degree in females than in males, while positive correlations were found for vascular markers, ICAM-1, VCAM-1, body mass index (BMI), and blood pressure [2]. Moreover, considering the reports that the risk of type II diabetes is positively correlated with the expressed serum levels of ICAM-1 and E-selectin and that a high-cholesterol diet could increase the serum concentrations of ICAM-1 and VCAM-1, CAMs should not be overlooked from the perspective of metabolic functions [3,4].

In greater detail, low-density lipoprotein cholesterol (LDL-C) accumulates in the lumen due to reasons such as hypertension, high blood cholesterol, and dyslipidemia, which flow into the tunica intima via damaged vascular endothelial cells, and are converted through oxidation into oxidized low-density lipoprotein (LDL) by reactive oxygen species (ROS). The excessive flow of LDL-C and the consequent formation of oxidized LDL in the arterial tunica intima lead to the conversion into and accumulation of foam cells, which marks the onset of vascular stiffness [5]. The level of oxidized LDL indicates not only the initial formation of arterial plaques, but also the risk of atherosclerotic cardiovascular disease [6]. Previous studies reporting high serum levels of oxidized LDL in patients with atherosclerosis at an early stage are supportive of the proposed clinical mechanism of vascular stiffness [7]. In addition, as vascular endothelial cells react sensitively in a severe state of inflammation with vascular aging, vascular stiffness in the elderly is accelerated by the induction of CAMs, which increases the adhesion of monocytes [8].

Thereby, vascular stiffness could be accelerated or aggravated when the reactivity of CAMs and oxidized LDL increases inordinately due to aging of vascular endothelial cells and, consequently, reduced functions. As vascular endothelial cells play key roles in vascular health, it is essential that pathological mechanisms related to vascular aging and stiffness be managed to prevent conversion into a pathological state.

Thus, exercise as an intervention with few side effects on physiological changes in the body is recommended for the elderly to be performed on a regular basis, which highlights the value of determining the intensity of suitable exercise and developing effective exercise programs. Changes in CAMs during exercise are influenced by exercise type or intensity, and when patients with cardiovascular disease are guided to perform aerobic exercise of low-to-moderate intensity, the expression levels of CAMs are reduced [9]. Moreover, regular exercise through a combination of aerobic and anaerobic training of moderate intensity caused positive changes in the oxidized LDL in obese elderly women, while the changes in skeletal muscles and the carotid artery medial thickness and oxidized LDL demonstrated a negative correlation [10]. These results collectively suggest that as a method to delay and conserve the functions of vascular endothelial cells, elderly women should perform moderate to high exercise to improve chronic inflammation caused by excess LDL-C accumulation in the tunica intima, which would downregulate the expression of CAMs and conversion of oxidized LDL into foam cells to improve and prevent vascular stiffness.

Thus, it is necessary to investigate the long-term effect of regular aerobic exercise of moderate-to-high intensity on the carotid to femoral pulse wave velocity (cf-PWV), CAMs, and oxidized LDL in elderly Korean women, and to verify the rate of change of vascular stiffness and the effect of and correlations across the variables regarding the rate of change.

## 2. Methods

### 2.1. Participants

The sample size in this study was estimated to be *n* = 36 using G-Power 3.1, based on the repeated measures analysis of variance and under the following conditions: effect size f = 0.28, group number *n* = 3, repeated measure *n* = 2, testing power at 0.8, and significance level at 0.05. We recruited female subjects aged 70–85 by placing recruitment notices at the senior community center, inviting individuals to voluntarily join our exercise program. We recruited 64 participants, considering a dropout rate of over 30%. For the group assignment criteria, subjects with a cf-PWV of 12 m/s or higher, who did not meet the obesity standards (BMI ≥ 25 kg/m^2^ and %BF ≥ 30%), were placed in the vascular stiffness group. Additionally, the obesity group included subjects who met the obesity standard but did not meet the cf-PWV threshold of 12 m/s or higher [11,12]. The normal group consisted of elderly women who did not meet either the cf-PWV or obesity standards.

**Figure 1:**
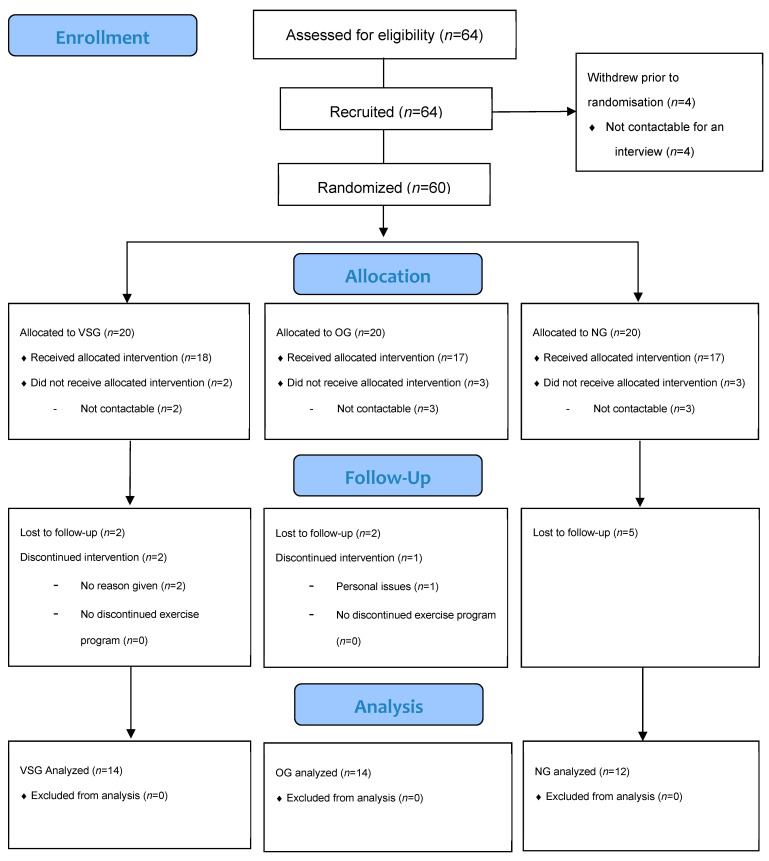
Procedures of this study.

After excluding participants who dropped out of the study during the exercise program due to personal reasons the total number of participants was *n* = 40. We then categorized them into three groups: the vascular stiffness group (VSG, *n* = 14), obesity group (OG, *n* = 14), and normal group (NG, *n* = 12). The measurements and test results of these groups were subsequently analyzed. Prior to the study, approval was obtained from the Pusan National University Institutional Review Board (PNU IRB/2022_44_HR). Detailed explanations of the study purpose and goals were provided to the participants, and signed consent was obtained from those who voluntarily agreed to participate. The study procedures and the physical characteristics of the participants are presented in Table 1 and Figure 1.

### 2.2. Aerobic Exercise Program

**Figure 2:**
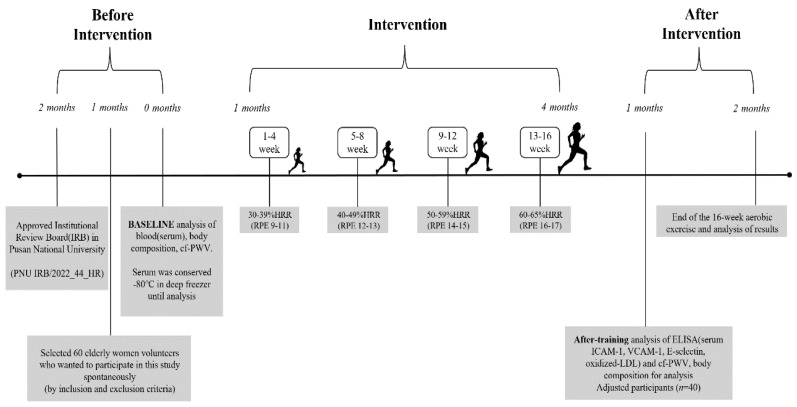
Flowchart of intervention.

For the aerobic exercise program in this study, the circuit exercise on functional fitness was revised and modified to develop a 16-week program of aerobic exercise at 60 min per session and three sessions per week [13]. The exercise intensity was based on the guidelines of the American College of Sports Medicine (ACSM) as follows [14]: 30–39% HRR (RPE 9-11) for weeks 1–4, 40–49% HRR (RPE 12-13) for weeks 5–8, 50–59% HRR (RPE 14-15) for weeks 9–12, and 60–65% HRR (RPE 16-17) for the final weeks, 13–16 [14], while a wireless heart rate sensor (Polar system, Finland) was used. The detailed procedures of aerobic exercise are presented in Figure 2.

## 3. Data Collection

### 3.1. Body Composition

The height (cm) of the participants was measured using a digital scale, and the weight (kg), body fat (%), and skeletal muscle mass (kg) were measured using an automatic scale Inbody 430 (Inbody, Seoul, Republic of Korea). Before undergoing Inbody measurements, subjects refrained from engaging in moderate-to-high intensity exercise for 12 h, and they also avoided consuming any food or beverages for 4 h.

### 3.2. Blood Collection

For the blood test, the participants were instructed by a clinical pathologist to maintain a fasting state from 8 PM on the day before the test, and between 8 and 10 AM on the day of the test, a vacutainer and needle were used to collect 10 mL of blood from the antebrachial vein. The collected blood was placed in a serum separator tube. After 20 min of centrifugation at 3000 rpm using Combi-514R (Hanil, Gimpo, Republic of Korea), the serum was isolated, and the supernatant was transferred to a 1.5-mL micro tube for storage at −80 °C for subsequent analyses.

### 3.3. Vascular Stiffness Analysis

To measure central blood pressure and analyze pulse wave and pulse wave velocity (PWV), each participant was instructed to rest in a supine position for 5 min, and vascular stiffness was measured using SphygmoCor XCEL (AtCor Medical Pty Ltd., Sydney, Australia), a tonometry device. In addition, for PWV, the time variation between measurements in the carotid and femoral arteries and the vascular length were estimated to express the PWV for the aorta as the cf-PWV.

### 3.4. Blood Analysis

Through enzyme-linked immunosorbent assay (ELISA), CAMs were analyzed using the human ICAM-1/CD54 allele-specific, human VCAM-1/CD106, and human E-selectin/CD62E of the R&D system at 450 nm absorbance using a microplate reader (Allsheng, Hangzhou, China). Oxidized LDL was also analyzed through ELISA by Mercodia oxidized LDL ELISA kit (Mercodia, Uppsala, Sweden) and at 450 nm absorbance using a microplate reader (Allsheng, Hangzhou, China).

### 3.5. Data Analysis

All data in this study were analyzed using SPSS Ver. 23.0. For each measured item, the mean (M) and standard deviation (SD) were estimated by group and factor. For the group × time interaction, two-way repeated measures analysis of variance (ANOVA) was used. One-way ANOVA was used for between-group variations. For the post hoc test, the *Scheffe* test was performed. Correlation analysis was based on Pearson correlation coefficients, and a simple regression analysis was performed to verify the effects of the variables. The level of significance was set at 0.05 for each item.

## 4. Results

### 4.1. Changes in cf-PWV

The cf-PWV showed a group × time interaction for VSG, OG, and NG (*p* < 0.001). The pre- to post-intervention between-group variation was significant for the VSG, OG, and NG (*p* < 0.01). The within-group variation over time was significant for the VSG (*p* < 0.01). Table 2 presents the changes in the cf-PWV examined in this study.

### 4.2. Changes in CAM

#### 4.2.1. ICAM-1

ICAM-1 showed no group × time interaction for VSG, OG, and NG, whereas significant within-group variation by time was found for VSG (*p* < 0.01) and NG (*p* < 0.05). Table 3 presents the changes in ICAM-1 levels examined in this study.

#### 4.2.2. VCAM-1

VCAM-1 showed a group × time interaction for the VSG, OG, and NG (*p* < 0.01). The within-group variation by time was significant for VSG (*p* < 0.001), and the rate of change also significantly varied across groups (*p* < 0.05). Table 3 presents the changes in VCAM-1 levels examined in this study.

#### 4.2.3. E-Selectin

E-selectin showed a group × time interaction for the VSG, OG, and NG (*p* < 0.05). The within-group variation by time was significant for VSG (*p* < 0.001), and the rate of change also significantly varied across groups (*p* < 0.01). Table 3 presents the changes in E-selectin levels examined in this study.

### 4.3. Changes in Oxidized LDL

Oxidized LDL in this study showed a group × time interaction for VSG, OG, and NG (*p* < 0.001). The within-group variation by time for VSG was significant (*p* < 0.01). The rate of change also varied significantly across groups (*p* < 0.001). The changes in oxidized LDL levels are presented in Table 4.

### 4.4. Correlation of the Rate of Change of cf-PWV with VCAM-1 and Oxidized LDL

**Figure 3:**
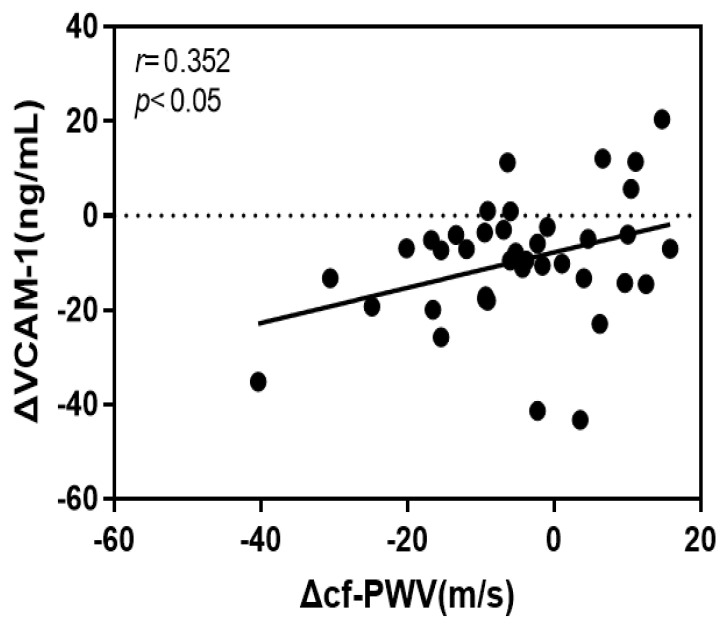
Scatter plots for pre–post exercise intervention change ration in cf-PWV and oxidized LDL.

**Figure 4:**
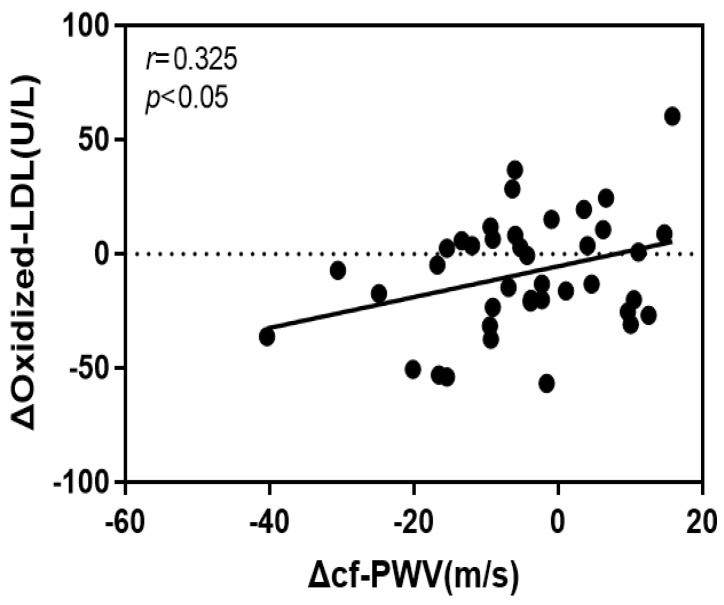
Scatter plots for pre–post exercise intervention change ration in cf-PWV and VCAM-1.

The rate of change of cf-PWV in this study had a significant positive correlation with that of VCAM-1 (*r* = 0.352, *p* < 0.05) and oxidized LDL (*r* = 0.325, *p* < 0.05). Figure 3 and Figure 4 show the respective scatter plots.

### 4.5. Analysis of the Effects of cf-PWV, VCAM-1, and Oxidized LDL

To determine the effect of the rate of change of cf-PWV on the rate of change of VCAM-1, the variables were tested, and a significant effect was observed. The coefficient of determination in the regression analysis was 0.124, indicating that 12.4% of the tested variables fit the standard regression line.

In addition, the variables for the effect of the rate of change of cf-PWV on the rate of change of oxidized LDL were tested, and the effect was shown to be significant. The coefficient of determination in the regression analysis was 0.106, indicate that 10.6% of the tested variables fit the standard regression line. Table 5 presents the results.

## 5. Discussion

In this study, the effects of a 16-week aerobic exercise program on elderly Korean women were evaluated. The intensity of the exercise was gradually increased, and the level of VCAM-1 was shown to be significantly reduced in elderly Korean women in the VSG, suggesting that the intervention of regular aerobic exercise could not only reduce the risk of heart failure, but also serve as an effective noninvasive treatment. In addition, the rate of change of cf-PWV was positively correlated with that of VCAM-1, and following exercise intervention, the reduction rates of cf-PWV and VCAM-1 were both high. In line with this, the results of this study verified that only the rate of change of VCAM-1 across CAMs could have an effect on the rate of change of the cf-PWV. Hence, VCAM-1 is thought to be most closely associated with the risk of vascular stiffness in elderly Korean women, and long-term regular exercise for 16 or more weeks is anticipated to be an effective method to improve vascular stiffness in the elderly.

As shown, the variation between VCAM-1 and ICAM-1 after the intervention could be attributed to the fact that the information conveyed by the quantified expression levels of the two molecules ICAM-1 and VCAM-1 was divided into two. For ICAM-1, the expression is simply a natural part of the inflammatory response, whereas VCAM-1 is expressed in a vascular environment with narrow arteries of low elasticity, which is thought to underlie the interaction effect observed solely for VCAM-1.

The clinical significance of these results is high for VCAM-1 in terms of vascular stiffness. Notably, as the adhesion of monocytes to the cell surface is promoted for their permeation to the endothelium, an increase in their expression could increase the surface permeability of endothelial cells, causing various lipids to accumulate in the extracellular matrix or lead to the dispersion of smooth muscle cells toward the accumulation of vascular plaques [15].

Exercise, however, enhances vascular function and causes the secretion of nitric oxide to increase vascular bioavailability to improve the ratio of the vascular wall to the lumen and the vascular diameter, which results from a positive effect of exercise-related hemodynamic stimulation on the vascular wall [16].

Therefore, when vascular endothelial cells are damaged, the risk of cardiovascular disease in sedentary elderly increases, whereas in the elderly with a habit of regular aerobic exercise, vascular endothelial cells can have enhanced functions. Moreover, cellular aging is prevented so that the functions of vascular endothelial cells could be conserved [17].

In a previous study on aerobic exercise, cardiac afterload was shown to decrease at rest or during exercise, as the increased cardiac output allowed adaptation to the exercise situation regarding vascular conductivity, resistance, blood flow conduction, and microvasculature, which could improve the ventricular functions to contribute to vascular health in the elderly [18].

Similarly, in this study, as the elderly Korean women without regular physical activities in the recent past three months acquired the habit of regular exercise at three times a week for 16 weeks by participating in the study, the functions of various molecules expressed in vascular endothelial cells could recover their functions, and such results are presumed to have had a positive effect on CAMs.

Further, as endothelial-derived particles are activated by high blood glucose concentrations, the expression of E-selectin could be increased by 30%, compared to the normal state in endothelial cells, and this overexpression caused the accumulation of various precipitates including lipids in the vessel, increasing the risk of vascular disease [19].

The pre-test results in this study also indicate an approximately 30% difference in expression when VSG, OG, and NG were compared, which implies that the regulation of E-selectin expression is important in both obesity and vascular stiffness. In addition, the significant changes observed in VSG after the intervention of regular exercise are thought to be significant regarding the functional improvement of endothelial cells, and the effect of reducing E-selectin has been verified further as another mechanism by which regular exercise could manage blood pressure.

By contrast, the OG in this study did not show significant differences in vascular stiffness and CAMs during the time before and after exercise, presumably due to the lack of improvement in vascular stiffness in obese individuals through aerobic exercise. Thus, a low BMI could have a stronger effect on the changes in PWV. In a meta-analysis of a previous study, the effect of aerobic exercise in improving vascular stiffness was also stronger in those with low BMI than in those with high BMI [20].

Similarly, in this study, the OG comprising the obese elderly Korean women with mean BMI ≥ 25 kg/m^2^ exhibited no significant effect, in support of a previous study. In other words, individuals with a high BMI, such as those with obesity, are not likely to benefit from regular exercise with respect to the positive effect on vascular stiffness. Hence, it would be more effective for these individuals to lose weight to manage their BMI and prevent vascular stiffness.

Further, a previous study comparing the vascular stiffness between a high-fat diet and a low-fat diet also showed that the stiffness was more severe in the aortic arch than in the descending aorta for a high-fat diet. Vascular endothelial cells in the area of arteries where a state of disturbed flow is created could exhibit a far higher level of stiffness in the process of increasing the absorption capacity of oxidized LDL [21], compared to those in a state of luminal flow. Hence, when shear stress decreases due to disturbed flow, the resulting leak in the area connecting the cells increases LDL permeability, ultimately increasing the probability of atherosclerotic plaques [22].

For this reason, the trend of decrease for VSG with respect to vascular stiffness and oxidized LDL was higher than that for OG, in contrast to the prediction that greater changes in oxidized LDL would be observed for OG as a result of aerobic exercise.

Based on the results, the positive correlation between the rate of change of cf-PWV and that of oxidized LDL in this study, as well as the effect of the rate of change of cf-PWV on that of oxidized LDL, could be attributed to the improved luminal flow from the disturbed flow in the elderly. In addition, a reason for the significant reduction in cf-PWV and oxidized LDL in the VSG after the 16-week aerobic exercise program could be the improvement in vascular stiffness in the aortic arch. It is considered that as vascular elasticity steadily decreases due to aging in the elderly, regular exercise to manage vascular stiffness is critical.

According to Boeno et al. [23], the intensity of aerobic exercise from 60%HRR to 80%HRR could improve the level of oxidized LDL without weight loss or BMI reduction, and the risk factors of cardiovascular disease were reduced. Similarly, the results in this study indicate that despite the administration of drugs for hyperlipidemia or to control blood pressure in the VSG, the gradually increased intensity of aerobic exercise led to an intensity ≥ 60%HRR with the beneficial effect on oxidized LDL. Thus, long-term regular aerobic exercise with an intensity of 30–65%HRR improved vascular health in elderly Korean women, and quality of life was shown to increase with increasing exercise duration and decreasing drug burden.

This study proposes that healthy vascular endothelial cells preserve the stability of the vascular structure by releasing active molecules, including those acting in an autocrine, paracrine, and endocrine manner, in response to both physical and chemical stimuli. Consequently, to support various vital physiological functions such as vascular tension, diameter regulation, smooth muscle cell proliferation, platelet function, and leukocyte adhesion, it is recommended that future research concentrates on enhancing substances linked to cardiovascular health, such as vascular stiffness and oxidized LDL, using non-invasive approaches.

Recognizing several limitations in this study, it’ is crucial to emphasize that we could not regulate the subjects’ daily activities, and the sample size was relatively small. Therefore, it may be challenging to extend the study’s findings to broader populations. Further research is needed to confirm our results, analyze the impact of aerobic exercise on variables in both obese and non-obese individuals, as well as in different age groups or exercise types for both men and women. Moreover, there is a need to design effective, age-specific aerobic exercise programs.

The findings of this study indicate that aerobic exercise is particularly beneficial for elderly women with elevated levels of vascular stiffness. However, to ensure the reliability of these results, further research is required. We anticipate comparative studies investigating various exercise intensities to strengthen the evidence for the effects of aerobic exercise. Moreover, we look forward to research pinpointing the most appropriate exercise intensity for elderly women, which can be achieved by assessing the acute expression of adhesion molecules. Additionally, we expect studies to comprehensively examine the relationship between aerobic exercise performance, adhesion molecules, and oxidized LDL in post-menopausal women experiencing a rapid progression of vascular stiffness.

## Figures and Tables

**Table 1:** Physical characteristics of subjects in each group.

Variables Group	Age (Years)	Height (cm)	Weight (kg)	BMI (kg/m^2^)	%BF (%)	cf-PWV (m/s)
VSG (*n* = 14)	78.64 ±4.55	152.39 ±4.71	51.48 ±6.70	22.13 ±2.12	29.26 ±5.56	14.77 ±1.94
OG (*n* = 14)	76.50 ±4.82	150.14 ±4.22	61.97 ±7.43	27.40 ±2.35	39.04 ±4.78	10.89 ±0.98
NG (*n* = 12)	78.75 ±5.79	147.83 ±6.70	48.55 ±7.06	22.14 ±2.03	30.08 ±6.34	9.94 ±1.31
*F*			13.331 ***	26.535 ***	13.107 ***	40.581 ***
*Scheffe*	*NS*	*NS*	VSG, NG, <OG	VSG, NG <OG	VSG, NG <OG	OG, NG <VSG

Values are M ± SD; BMI: body mass index, %BF: percentage of body fat; cf-PWV: carotid to femoral pulse wave velocity; VSG: vascular stiffness group, OG: obesity group, NG: normal group; *** *p* < 0.001, *NS*: non-significant.

**Table 2:** Changes in cf-PWV after 16-week aerobic exercise program.

Variable	Group	Pre	Post	*diff*(%)	*T*	*F*	
cf-PWV (m/s)	VSG (*n* = 14)	14.77 ±1.94	12.61 ±1.73	−14.62	3.712 **	Group	31.127 ***
	OG (*n* = 14)	10.89 ±0.98	10.88 ±1.30	−0.09	0.058	Time	9.422 **
	NG (*n* = 12)	9.94 ±1.31	9.93 ±1.40	−0.10	0.052	G × T	9.319 ***
	*F*	40.581 ***	10.983 ***	7.730 **			
	*Scheffe*	OG, NG <VSG	OG, NG <VSG	NG < OG <VSG			

Values are M ± SD; cf-PWV: carotid to femoral pulse wave velocity; VSG: vascular stiffness group, OG: obesity group, NG: normal group; ** *p* < 0.01, *** *p* < 0.001.

**Table 3:** Changes in cell adhesion molecules after 16-week aerobic exercise program.

Variable	Group	Pre	Post	*diff*(%)	*t*	*F*	
ICAM-1 (ng/mL)	VSG (*n* = 14)	10.57 ±2.89	9.48 ±2.45	−10.31	3.493 **	Group	0.804
	OG (*n* = 14)	9.92 ±3.69	9.62 ±2.75	−3.02	0.674	Time	13.462 ***
	NG (*n* = 12)	11.84 ±4.55	10.75 ±3.33	−9.20	2.740 *	G × T	1.404
	*F*	0.873	0.760	2.898			
	*Scheffe*	*NS*	*NS*	*NS*			
VCAM-1 (ng/mL)	VSG (*n* = 14)	33.15 ±6.65	26.94 ±4.84	−18.73	5.241 ***	Group	1.745
	OG (*n* = 14)	28.13 ±5.81	25.67 ±4.20	−8.75	2.144	Time	28.254 ***
	NG (*n* = 12)	29.29 ±3.67	28.11 ±2.41	-4.03	1.659	G × T	6.006 **
	*F*	3.060	1.197	5.115 *			
	*Scheffe*	*NS*	*NS*	NG < OG <VSG			
E-selectin (ng/mL)	VSG (*n* = 14)	4.74 ±1.37	3.71 ±1.50	−21.73	4.236 ***	Group	1.258
	OG (*n* = 14)	4.42 ±2.04	4.22 ±1.42	−4.52	0.816	Time	11.157 **
	NG (*n* = 12)	3.57 ±1.30	3.39 ±1.28	−5.04	0.771	G × T	4.052 *
	*F*	1.766	1.143	5.389 **			
	*Scheffe*	*NS*	*NS*	OG < NG <VSG			

Values are M ± SD; ICAM-1: intracellular cell adhesion molecule-1; VCAM-1: vascular cell adhesion molecule-1; E-selectin: endothelial selectin; VSG: vascular stiffness group, OG: obesity group, NG: normal group; * *p* < 0.05, ** *p* < 0.01, *** *p* < 0.001, *NS*: non-significant; G × T: between group and time interaction.

**Table 4:** Changes in oxidized LDL after 16-week aerobic exercise program.

Variable	Group	Pre	Post	*diff*(%)	*t*	*F*	
Oxidized LDL (U/L)	VSG (*n* = 14)	10.40 ±3.22	7.38 ±2.99	−29.04	4.145 **	Group	0.235
	OG (*n* = 14)	8.69 ±3.33	8.67 ±2.87	−0.23	0.027	Time	11.895 ***
	NG (*n* = 12)	8.32 ±2.98	7.96 ±2.72	−4.33	0.730	G × T	8.740 ***
	*F*	1.630	0.712	8.920 ***			
	*Scheffe*	*NS*	*NS*	OG, NG < VSG			

Values are M ± SD; Oxidized LDL: oxidized low-density lipoprotein; VSG: vascular stiffness group, OG: obesity group, NG: normal group; ** *p* < 0.01, *** *p* < 0.001, *NS*: non-significant; G × T: between group and time interaction.

**Table 5:** Simple regression analysis of cf-PWV change ratio and VCAM-1/oxidized LDL change ratio following 16-week aerobic exercise program.

Dependent Variable	B	SE	β	*t*	*F*	*R* ^2^
Constant	−7.723	2.110		−3.660 ***		
VCAM-1	0.374	0.162	0.352	2.315 *	5.359 *	0.124
Oxidized LDL	0.675	0.319	0.325	2.118 *	4.485 *	0.106

* *p* < 0.05, *** *p* < 0.001.

## Data Availability

Not applicable.
